# Plant community feedbacks and long-term ecosystem responses to multi-factored global change

**DOI:** 10.1093/aobpla/plu035

**Published:** 2014-07-14

**Authors:** J. Adam Langley, Bruce A. Hungate

**Affiliations:** 1 Department of Biology, Villanova University, Villanova, PA 19085, USA; 2 Center for Ecosystem Science and Society, Deparment of Biological Sciences, Northern Arizona University, Flagstaff, AZ 86011, USA

**Keywords:** CO_2_ fertilization, ecological tradeoffs, elevated CO_2_, multiple factors, nitrogen pollution, plant productivity

## Abstract

While short-term plant responses to global change are driven by physiological mechanisms, which are represented relatively well by models, long-term ecosystem responses to global change may be determined by shifts in plant community structure resulting from other ecological phenomena such as interspecific interactions, which are represented poorly by models. In single-factor scenarios, plant communities often adjust to increase ecosystem response to that factor. For instance, some early global change experiments showed that elevated CO_2_ favours plants that respond strongly to elevated CO_2_, generally amplifying the response of ecosystem productivity to elevated CO_2_, a positive community feedback. However, most ecosystems are subject to multiple drivers of change, which can complicate the community feedback effect in ways that are more difficult to generalize. Recent studies have shown that (i) shifts in plant community structure cannot be reliably predicted from short-term plant physiological response to global change and (ii) that the ecosystem response to multi-factored change is commonly less than the sum of its parts. Here, we survey results from long-term field manipulations to examine the role community shifts may play in explaining these common findings. We use a simple model to examine the potential importance of community shifts in governing ecosystem response. Empirical evidence and the model demonstrate that with multi-factored change, the ecosystem response depends on community feedbacks, and that the magnitude of ecosystem response will depend on the relationship between plant response to one factor and plant response to another factor. Tradeoffs in the ability of plants to respond positively to, or to tolerate, different global change drivers may underlie generalizable patterns of covariance in responses to different drivers of change across plant taxa. Mechanistic understanding of these patterns will help predict the community feedbacks that determine long-term ecosystem responses.

## Abstract (precis)

If you add CO_2_ or nitrogen to a single plant it will likely grow more, but the amount by which each resource stimulates growth differs widely across species. When you add either resource to a whole ecosystem, total plant growth will likely also increase, but there will be winners and losers, causing a change in the relative abundance of plant species, and therefore altering the way the whole ecosystem responds to the added resource, a “community feedback”. These feedbacks are very difficult to predict, especially when multiple resources are added, but a lot of recent experimental evidences suggests that community feedbacks will determine how future ecosystems operate.

## Introduction

Unpredictability in the functioning of terrestrial ecosystems underlies large uncertainties in predictions of future atmospheric CO_2_ content (Friedlingstein *et al.* 2006; Denman *et al.* 2007; Matthews 2007; Dolman *et al.* 2010; Piao *et al.* 2013). The uncertainty among projections of the terrestrial carbon sink over the next century is equivalent to 50 years of greenhouse gas emissions at current rates (Sitch *et al.* 2008). Some Earth System models incorporate biogeochemical constraints such as those imparted by the nitrogen (N) cycle on plant productivity and respiration and thus ecosystem carbon exchange (Thornton *et al.* 2007; Sokolov *et al.* 2008; Bonan and Levis 2010; Bala *et al.* 2013), but these models may still overestimate the ability of ecosystems to offset human emissions (Piao *et al.* 2013). Long-term carbon balance may be sensitive to other, slow-acting ecological feedbacks (Friedlingstein and Prentice 2010) that affect the magnitude of the CO_2_ fertilization effect in terrestrial ecosystems.

Though Earth System models have incorporated more sophisticated dynamic global vegetation models to represent vegetation shifts, relatively short-term, physiological mechanisms still underpin plant responses (Smith and Dukes 2013). A major source of uncertainty in these models is their ability to predict changes in plant communities in ways that exert a strong influence on ecosystem response to global change (Scheiter *et al.* 2013), recently referred to as the ‘community effect’ (Polley *et al.* 2014). Plant community shifts in response to global changes could initiate key feedbacks that ultimately overwhelm short-term physiological responses (Chapin *et al.* 1997; Shaver *et al.* 2000; Suding *et al.* 2008; Smith *et al.* 2009). Moreover, the community shifts observed in global change experiments may not correspond to those expected based on short-term physiochemical responses of individual species or functional groups (e.g. Kardol *et al.* 2010; Langley and Megonigal 2010; Niu *et al.* 2010; Dawes *et al.* 2013; Isbell *et al.* 2013; Smith *et al.* 2013; Ward *et al.* 2013).

Previous theoretical work has established how changes in plant community structure (defined herein as changes in species identity, richness or evenness), either from shifts of dominance within an ecosystem, immigration or extinction, may drive ecological feedbacks on long-term time scales (Smith *et al.* 2009) that are relevant for global change predictions. Here, we apply this idea to multi-factored change by (i) surveying recent literature that highlights the importance of community dynamics in long-term, multi-factor field manipulations, (ii) presenting new data from a brackish marsh as a case study, and (iii) using a simple community-net primary production (NPP) model to illustrate how community shifts can mediate ecosystem responses to multiple global changes. We emphasize the marsh as a case study because the plant community is simple (richness = 3) and dynamic so that it responds rapidly to new conditions. The responses observed in the marsh may therefore illustrate mechanisms that apply in other ecosystems that have more complex communities such as grasslands, or ecosystems that require much more time to equilibrate, such as forests. We emphasize the challenge of predicting the responses of future ecosystem productivity to elevated CO_2_ in combination with other drivers; however, the concepts may be generally applicable to other combinations of drivers, as well as other ecosystem responses.

## Community Effects in Response to Single Factors

Single-factor scenarios provide a simple starting point to evaluate the importance of the community effect. Rising CO_2_ should shift communities towards dominance by plant species that respond most positively to elevated CO_2_ (Purves and Pacala 2008; Polley *et al.* 2012). Typically, such a unidirectional community response will have an amplifying effect on the ecosystem response and an ameliorating effect on the initial resource perturbation. For instance, models that allow for vegetation dynamics yield stronger negative feedbacks on atmospheric CO_2_ rise than those that have static communities (Purves and Pacala 2008). As species that respond strongly to CO_2_ increasingly dominate, enhanced ecosystem CO_2_ uptake will tend to counteract the resource perturbation, which in this case is rising atmospheric CO_2_ concentrations.

Examples from the first generation of global change experiments showed in single-factor studies that plant community structure responded predictably to one perturbation and had predictable consequences for ecosystem processes. For instance, N addition strongly shifted plant dominance towards nitrophilic species, those with a greater capacity to take advantage of extra N, enhancing total ecosystem productivity in a Minnesota grassland (Tilman 1987). In a brackish marsh, 4 years of exposure to elevated CO_2_ yielded increasing plant dominance by C_3_ plants over C_4_ grasses in mixed plots, enhancing the overall ecosystem response (Arp *et al.* 1993). These types of findings supported the idea that plant community shifts tend to strengthen ecosystem response to resource addition.

However, as global change studies increased in duration, encompassing more background environmental variability and others included multiple factors, the responses became more idiosyncratic. After 18 years of exposure, the same brackish marsh plots showed a weak response of plant community to elevated CO_2_ compared with the community responses to background variability in other abiotic factors (Erickson *et al.* 2007). Similarly, elevated CO_2_ initially drove a shift in species dominance in a New Zealand grassland, but after 11 years, an additional factor of grazing negated that shift and the ecosystem CO_2_ response (Newton *et al.* 2014).

The short- and long-term effects of global change drivers may differ for several reasons. For instance, the effects on vegetative production may differ from effects on reproduction or recruitment (including floral production, seed production, seed quality, seedling establishment), leading to longer-term demographic shifts that are not predictable from short-term growth responses (Williams *et al.* 2007). Moreover, the future will bring multiple changes simultaneously, and some factors, like N eutrophication, may have stronger effects on plant community structure than others, like rising CO_2_ (Reich 2009). What happens to the community feedback when multiple factors important to plant physiology and growth change at once?

## Community Shifts in Response to Multiple Factors

Relatively few long-term, *in situ* global change experiments have manipulated multiple factors (Norby and Luo 2004), but several have recently found that the combined effect on ecosystems differs substantially from the sum of single-factor effects (Table 1). For example, a global change experiment in a Californian grassland found a CO_2_ stimulation of plant growth when CO_2_ was applied in isolation but not in combination with warming or N addition (Shaw *et al.* 2002; Dukes *et al.* 2005), and similarly, CO_2_ effects on N_2_O emissions were dampened with other factors (Brown *et al.* 2012). In a hardwood forest, high CO_2_ enhanced growth but not in combination with ozone (Karnosky *et al.* 2003) or climatic variability (Kubiske *et al.* 2006). In a tidal marsh, the CO_2_ stimulation of productivity diminished with added N (Langley and Megonigal 2010). More generally, the magnitude of global change responses decreases with an increasing number of drivers (Leuzinger *et al.* 2011).

**Table 1.** Long-term, multifactor, CO_2_ studies that report on plant community feedbacks.

Site	CO_2_ treatment	Other treatments	Duration (years)	CO_2_ effects on NPP	Plant community response to CO_2_ only and/or effects on response	Multiple-factor community feedbacks	Citation
MN grassland BIOCON	Ambient + 180 ppm	N	13	13 % (R&H, 2012)	CO_2_ increased biodiversity, yielding greater CO_2_ stimulation	N addition enhanced CO_2_ response of ANPP. Interactive effects independent of community richness or composition	Isbell *et al.* (2013), Reich and Hobbie (2012)
Swiss pasture	Ambient + 240 ppm	N	10	Increased belowground but not AGB. Decreased N concentration	More *T. repens* at elevated CO_2_ (33 % instead of 21 %) at expense of *L. perrene* (Hartwig *et al.* 2000)	High N addition enhanced CO_2_ response of AGB	Schneider *et al.* (2004), Hartwig *et al.* (2000)
MD Marsh	720 ppm	N	4	10 % over 4 years	No	N addition strongly shifted plant community towards C_4_ dominance, reversing CO_2_ effect	Langley and Megonigal (2010)
CA grassland Jasper Ridge	680 ppm	Temp, N, H_2_O	4	No significantly effect onshoot or root production	Forb abundance was lower (Zavaleta *et al.* 2003)	Adding warming or precipitation to CO_2_ treatment increased forb abundance, while adding N decreased it, no CO_2_ effect with *N*	Dukes *et al.* (2005), Zavaleta *et al.* (2003)
Tasmanian grassland	550 ppm	Temp	4	60 % increase in ANPP over 4 years	CO_2_ increased C_4_ dominance from 25 to 39 %	Warming decreased C_4_ dominance. No interaction reported	Pendall *et al.* (2011)
WY Prairie	600 ppm	Temp	3	Peak total AGB increased an average of 33 % (but not during wet year).	Favoured C_3_ grasses (34 % greater AGB than control) over C_4_ (28 % greater)	Warming favoured C_4_ grasses reducing CO_2_ effect	Morgan *et al.* (2011)
Danish heathland	510 ppm	Temp, H_2_O	3	No	Increased biomass of *D. flexuosa* (grass) in 1 of 3 years. No significant effect for *C. vulgaris* (evergreen dwarf shrub)	Less *D. flexuosa* biomass in warmed + drought plot compared with drought plots but CO_2_ counterbalanced this decrease in three-factor plots	Kongstad *et al.* (2012)
French upland grassland	600 ppm	Temp	3	No significant effect on AGB	No	Proportion of *Agrostris capillaris* increased in warm + drought + CO_2_ vs. warm + drought. Proportion of *Trisetum flavescens* decreased in warm + drought + CO_2_ compared with control	Bloor *et al.* (2010)
TX grassland	200–560 ppm range	H_2_O	4	35 % averaged over 4 years	Yes, accounted for 21–38 % of CO_2_ stimulation	Added precipitation altered community, but was not applied factorially	Polley *et al.* (2014)
New Zealand grassland	475 ppm	Grazing	11	Positive, but reported elsewhere?	CO_2_ decreased proportion of C_3_ grasses and increased legumes and forbs driving CO_2_ response	Grazing reduced proportion of legumes and forbs, allowing grasses to increase, eliminating CO_2_ response	Newton *et al.* (2014)

AGB, aboveground biomass; ANPP, aboveground net primary production.

**Figure 1.**
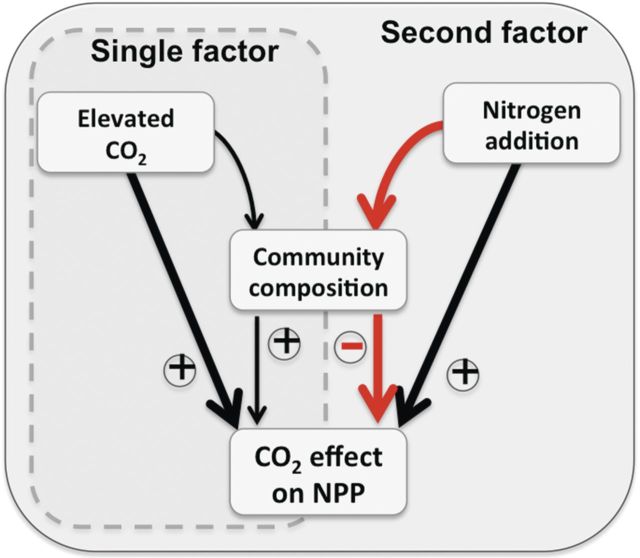
The pathways of influence by which elevated CO_2_ and N may drive an ecosystem function. Symbols represent the effect of added resources on each relationship. The black arrows represent short-term, physiological effects of N addition on the CO_2_ fertilization effect, which are generally found to be positive for individual plants. The red arrows represent the effect of N-driven community shifts, which may counteract or enhance the physiological effects depending on how they covary with the other pathways of influence. Elevated CO_2_ may also affect community change, but the effects are typically not as pronounced as with added N.

**Figure 2.**
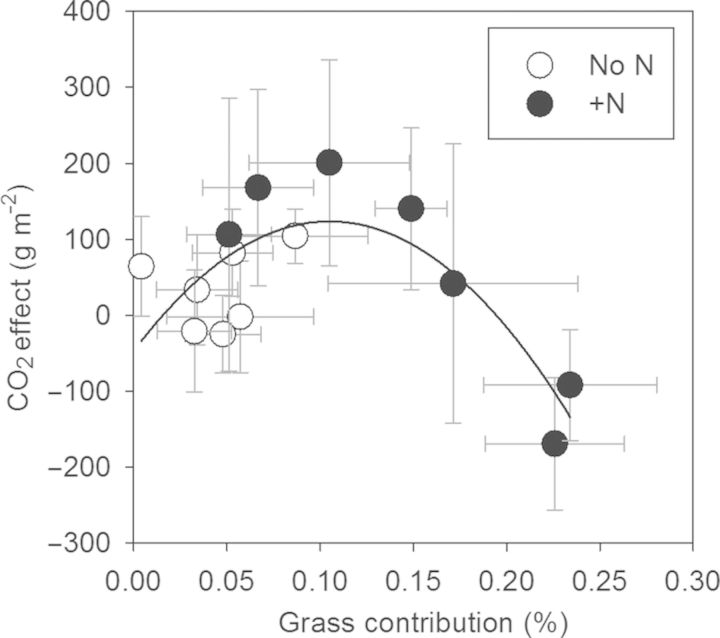
Annual NPP response to elevated CO_2_ (elevated NPP–ambient NPP) by annual grass contribution to community dominance (percent grass biomass in elevated CO_2_ treatment or elevated CO_2_ + N treatment for N-fertilized plots) from a tidal wetland. Data are grouped by N treatment (no N: white circles, added N: black circles) from 2006 to 2012. Methodological details are as described in Langley and Megonigal (2010); data presented here show additional years of the study (2010–12).

Plant community feedbacks may help explain this pattern in ecosystem response. Elevated CO_2_ alone tends to favour dominance by CO_2_-responsive species yielding a more CO_2_-responsive ecosystem (Arp *et al.* 1993; Newton *et al.* 2014; Polley *et al.* 2014), but addition of N can cause unexpected effects mediated by plant community shifts (Fig. 1; Langley and Megonigal 2010). While elevated CO_2_ and N addition tend to have direct and positive effects on the productivity of plants in isolation, each resource has different effects on plant communities by favouring different subsets of species. Moreover, N addition generally imparts a much more profound influence on the community than CO_2_ (Reich 2009; White *et al.* 2012). In the tidal marsh, N addition promoted dominance by C_4_ grasses that respond poorly to elevated CO_2_, thereby reducing ecosystem responsiveness (Fig. 1, second factor). In years when N had a strong effect on dominance by grasses, it also reduced the response of NPP to elevated CO_2_ (Fig. 2).

A new generation of decadal-scale global change studies is converging on the finding that the strongest effects of global change may be mediated by plant community shifts, eclipsing other more direct effects on plant physiology (Kardol *et al.* 2010; Langley and Megonigal 2010; Niu *et al.* 2010; Yang *et al.* 2011; Wu *et al.* 2012; Isbell *et al.* 2013; Newton *et al.* 2014; Polley *et al.* 2014). In a Tasmanian grassland, plant functional type had a large effect on ecosystem carbon cycling (Pendall *et al.* 2011). A recent analysis of N cycle response to warming and clipping found that the strongest effects of N on productivity were driven by species shifts (Niu *et al.* 2010). In an old-field ecosystem, Kardol *et al.* (2010) found that differences in soil processes varied more strongly among plant species than between any global change treatments such as watering, added N and elevated CO_2_, implying that in the long term, even small changes in species abundance would hold more sway than the sum of physiological changes imparted on individual species. Warming enhanced NPP in an arid grassland until community shifts negated the stimulation (Wu *et al.* 2012). Even though N addition strongly increased plant productivity in a Minnesota grassland initially, the effect diminished in the long term owing to changes in plant diversity (Isbell *et al.* 2013).

While community shifts may take decades to occur, direct physiological responses can occur within seconds but may diminish through time because of acclimation (Isbell *et al.* 2013; Smith and Dukes 2013). For instance, the short-term physiological response and ecosystem productivity response to CO_2_ often diminish in the longer term (Seiler *et al.* 2009; Lee *et al.* 2011). Likewise, warming of soil causes a short-term increase in respiration that wanes over time (Bradford *et al.* 2008). Therefore, while experiments are likely to overestimate the direct influence of global change, they also may underestimate community effects, particularly in plant communities that change slowly (Smith *et al.* 2009). Forest community dynamics are particularly difficult to capture owing to long individual lifespans. Tree species shifts in response to CO_2_ have not been adequately examined (Norby and Zak 2011), though chronic N pollution is beginning to alter tree species in some forests (e.g. Zaccherio and Finzi 2007).

Thus, the key to predicting long-term ecosystem responses and responses to multiple factors may lie in predicting long-term plant community changes. For instance, while a diminished ecosystem response to multiple factors may be partially explained by different treatments counteracting each other (i.e. warming may dry soil while water addition will restore moisture) (Luo *et al.* 2008; Leuzinger *et al.* 2011), mounting evidence indicates that in the long term, changes in plant community structure could ultimately govern ecosystem responses (Figs 1 and 2; Smith *et al.* 2009; Langley and Megonigal 2010; Niu *et al.* 2010). Yet, accurately predicting these phenomena requires understanding community responses mechanistically.

## Tradeoffs in Plant Responsiveness to Different Perturbations

A cornerstone of modern ecological theory is that plants exhibit tradeoffs in resource strategy (Tilman 1988; Craine 2009), such that optimization for acquisition and use of one resource precludes optimal acquisition and use of another. A simple example of a tradeoff is that of plant allocation among plant organs. Energetic limitations dictate that the same resources cannot be allocated simultaneously to multiple organs, while phylogenetic limitations appear to dictate that a single plant has limited plasticity in shifting allocation—that is, plant species are hardwired to a narrow range of allocation strategies (Reynolds and D'Antonio 1996; Müller *et al.* 2000; Craine and Dybzinski 2013). For example, N addition induced only small changes in plant allocation patterns compared with the inherent variability across 27 herbaceous species (Müller *et al.* 2000).

These differences in allocation strategy could help explain how plants respond to global changes, particularly those that alter resource availability. For instance, plants that exhibit low root-to-shoot biomass ratios are optimized for relatively high nutrient conditions, and should be better positioned to respond positively to soil resource addition than plants that have high root-to-shoot ratios. Other tradeoffs in physiology or allocation may play a role in resource strategy. For instance, a tradeoff may exist in plant metabolic pathways such that a positive response to elevated CO_2_ may interrupt the efficient incorporation of nitrate-N into a usable organic form (Bloom *et al.* 2010). If these tradeoffs result in a predictable relationship between the magnitude of plant growth response to elevated CO_2_ and that to added N, then this knowledge could help constrain our predictions of ecosystem responses to global change.

Evidence for a tradeoff between CO_2_ and N responses exists across functional groups. For instance, legumes respond most strongly to CO_2_ compared with other functional groups (Jablonski *et al.* 2002) and are among the weakest responders to N addition (Xia and Wan 2008). At the other end of the spectrum, grasses tend to exhibit a very weak CO_2_ response (Jablonski *et al.* 2002; Ainsworth and Long 2005), but respond strongly to N enhancement in many ecosystems (Pennings *et al.* 2002; Langley and Megonigal 2010). Slow-growing plants generally respond more positively than fast-growing ones to elevated CO_2_ (Ali *et al.* 2013), while the opposite is true for responses to N addition. Still, enormous variability exists within functional groups, making it difficult to generate useful functional group parameterizations for models. Again, these tradeoffs in response may occur among other resources or conditions as well. Recent evidence, for example, suggests differential responses to CO_2_ and water, and the same responses to CO_2_ and warming (Dijkstra *et al.* 2010; Dieleman *et al.* 2012).

## A Simple Plant Community Model

To examine the potential importance of plant community shifts for ecosystem response we simulated a simplified ecosystem with 30 species of initially equal biomass. We assigned realistic, randomized distributions of biomass responses to each plant species modelled after observed distributions of responses. Carbon dioxide response ratios (*R* = treated/control) across species are generally normally distributed with a mean of 1.25 and a standard deviation of 0.35 (Poorter and Navas 2003). Nitrogen stimulations are typically log-normally distributed (Xia and Wan 2008) with a mean of 1.29 and a standard deviation of 0.37 (LeBauer and Treseder 2008). (Note that model outputs are qualitatively insensitive to the effect sizes used.) We assigned a random value (*P*) from 0 to 1 to each of 30 species. We used *P* as a cumulative probability to calculate the corresponding CO_2_ response according to a normal distribution (*μ* = 1.25, *σ* = 0.35).

Then, we generated combined (CO_2_ + N) responses for three types of relationships between CO_2_ and N responses: negative, positive and independent, representing three ways that responses to each driver may covary. For ‘negative’, the randomly generated probability was inverted (1−*P*) to generate the N response of each species using a lognormal distribution (*μ* = 1.29, *σ* = 0.37). Therefore, a species with a large CO_2_ response would have a relatively small N response and *vice versa*. The negative relationship represents a tradeoff wherein plants that respond strongly to CO_2_ do not respond strongly to N. For ‘positive’, each species probability (*P*) was converted to an effect size for N using the lognormal distribution of N responses (*μ* = 1.29, *σ* = 0.37) such that a species that responds most strongly to CO_2_ also responds most strongly to N. A positive relationship could occur if the capacity to respond to both N and CO_2_ was related to some other factor. Some have suggested, for instance, that plants with high relative growth rates should be more suited to take advantage of increased resource availability, though this has not been observed for elevated CO_2_ (Poorter and Navas 2003). The ‘independent’ scenario assumes that the N response of a species is unrelated to the CO_2_ response, so random probabilities were generated independently for the N responses and then converted using the N response distribution (*μ* = 1.29, *σ* = 0.37). The response ratios were maintained for each species throughout the simulation.

We calculated a species biomass response ratio (*R*) for each species in each scenario. For single-factor scenarios (CO_2_ only and N only), we used *R* for each species based only on either CO_2_ or N responses. For the CO_2_ + N scenarios (independent, positive and negative) we calculated additive and multiplicative effects of CO_2_ and N. Additive was estimated as $R_{X}=R_{{\mathrm{CO}}_{2}}+R_{\mathrm{Nitrogen}}$. Multiplicative stimulation was calculated as $R_{X}=R_{{\mathrm{CO}}_{2}}\times R_{\mathrm{Nitrogen}}$. These two methods represent two ways CO_2_ and N may interact in ecosystems (Reich *et al.* 2006). Because the additive and multiplicative simulations yielded qualitatively similar responses, we limit further discussion to the multiplicative. We weighted each plant response according to its relative abundance (*A_X_*) to calculate total ecosystem biomass response (ER):

$$\text{ER}=\sum (A_{X}R_{X})$$

Therefore, the first-generation ecosystem response is equivalent to the average of individual plant responses because the distribution of species was initially even. For subsequent generations, we allowed species relative abundance in a given generation (*t*) to shift according to its relative biomass response in the previous generation (*t* − 1):

$$A_{X(t)}=R_{X(t-1)}/{\text{ER}}_{\left(t-1\right)}$$

Here we assumed that community shifts follow biomass responses. This assumption may not hold as, for instance, reproductive response, which may influence long-term demography, can differ from the growth response (Williams *et al.* 2007; Way *et al.* 2010). Still, there is evidence that long-term plant community shifts may respond consistently over time to some drivers like N eutrophication more strongly than to others like elevated CO_2_ (Reich 2009; Langley and Megonigal 2010). To account for the possibility that community shifts follow N responses but not the combined CO_2_ × N responses, we conducted a simulation using just the single-factor *R*_X_ representing the N response.

Because no species are added and none go extinct, the model represents physiological responses and shifts within the community, or species reordering (Smith *et al.* 2009), but not exchange with external species pools. However, species relative abundance is allowed to drop infinitesimally close to zero, so that species may effectively go extinct in some cases.

**Figure 3.**
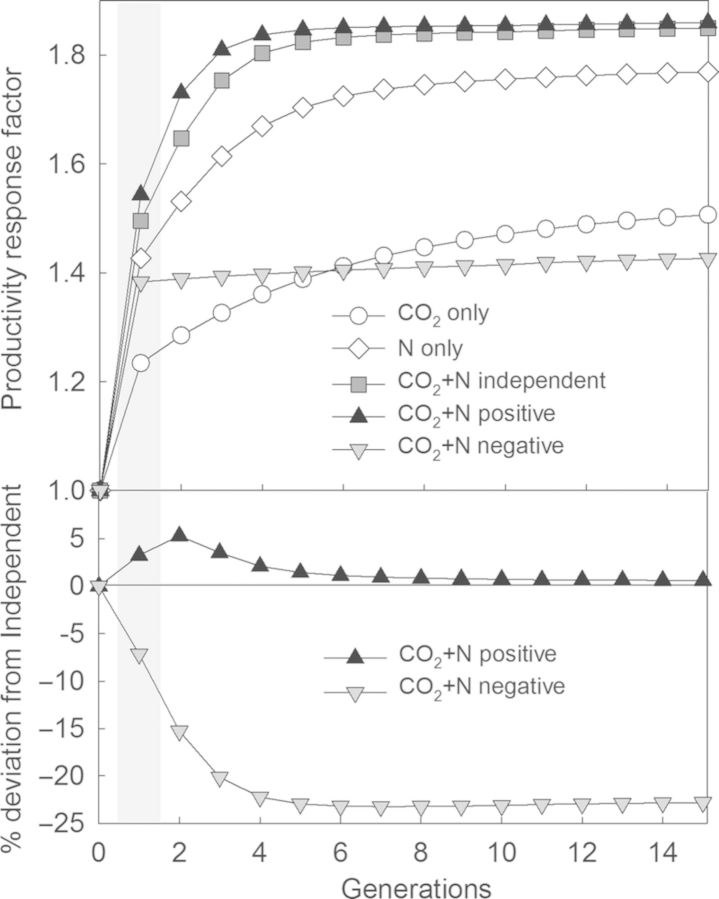
Ecosystem productivity response (top panel) and relative difference in productivity (bottom panel) for simulated plant communities for single factors (CO_2_ and N) and combined responses (CO_2_ + N) according to the relationships between CO_2_ response and N response across species (independent, positive or negative). Ecosystem productivity is expressed as a factor of the control with ambient CO_2_ and no added N, which is set to 1. The first-generation response (highlighted in grey box) represents the physiological effect while subsequent generations represent the influence of dynamic communities. For instance, while CO_2_ alone here yields a response factor of 1.24 in the first year, ensuing community shifts favouring CO_2_-responsive species could increase the response to 1.49 by year 2015.

To constrain the upper limit of ecosystem response we imposed a light limitation according to an established relationship between leaf area index (LAI) and canopy assimilation given a high light environment given the following equation (Bonan 2008):

$$A_{\mathrm{canopy}}=(A_{\max}/K)\times \text{ln }[(A_{\max}/\epsilon +F_{0})/(A_{\max}/\epsilon +F_{0}{\text{e}}^{\left(-KL\right)})]$$

where *A*_canopy_ is the ecosystem level CO_2_ assimilation per ground area used as a proxy for plant growth; *A*_max_ is the light saturated leaf-level assimilation (20 µmol m^−2^ s^−1^); ε is the light use efficiency (0.06); *K* is the light extinction coefficient (0.5); *F*_0_ is the solar radiation at the top of the canopy (1000 µmol m^−2^ s^−1^); and *L* is the LAI, which was set initially at 2.5 and allowed to vary through time. Then, we allowed shifts to occur for 14 additional generations to look at the trajectory of ecosystem response with shifting community structure (Fig. 3). We repeated the simulation with re-randomized response distributions 20 times to estimate mean response and error.

Though this exercise makes many simplifying assumptions, the results illustrate some important points. If the ultimate community shifts follow initial, short-term responses, then the community feedback effect will amplify individual physiological responses to an enhanced resource in isolation to other global change factors or other disturbances (‘CO_2_ only’ and ‘N only’ scenarios; Fig. 3, top panel). The ultimate ecosystem NPP response roughly doubles the initial stimulation, intuitively approaching the stimulation of the strongest responding species. This finding agrees with initial results from many ecosystem studies described above.

With multiple interacting factors, the magnitude of the initial ecosystem response (at Generation 1) and especially the community effect (apparent in subsequent generations) depends on how plant responses to one factor relate to that of another factor. The existence of a negative relationship between CO_2_ response and N response across plant species, which would arise if tradeoffs exist in the ability of plants to respond to enhancement of each resource, will reduce total ecosystem productivity in the long term below what would be predicted if CO_2_ and N responses are independent or positively related. Interestingly, the model indicates that with negative covariance, community feedbacks in response to CO_2_ and N in combination could lead to an ecosystem productivity stimulation that is smaller than with CO_2_ only (Fig. 3). The short-term responses do not depend as strongly on the type of covariance (from 42 to 56 % stimulation, Generation 1; Fig. 3) as the long-term responses (from 46 to 86 % stimulation, Generation 15; Fig. 3).

We have made the point above, however, that long-term community shifts do not always follow initial physiological responses. If the ultimate community shifts occur randomly and independently of the physiological stimulation, then the ultimate stimulations would resemble the initial stimulation (Generation 1), and community shifts would be unimportant for predicting long-term ecosystem response. It is more likely that community shifts *are* related to physiological responses to global change drivers, though the shifts may not occur in a manner as simple as represented in this model. For instance, N addition tends to have a stronger influence on community structure than does elevated CO_2_ (Reich 2009; Langley and Megonigal 2010). Therefore, we may expect that plant communities exposed to both CO_2_ and N would follow the initial N response more than the initial CO_2_ response. When community shifts followed N response, the results were dampened but qualitatively similar to when the community followed the combined CO_2_ × N response (results not shown).

The model indicates that failing to properly account for plant community shifts could engender large errors in estimates of long-term ecosystem productivity. In the case of multi-factored global change, the community feedback effect depends strongly on the type of covariance plant responses exhibit among different factors. For instance, failing to account for negative covariance between CO_2_ and N response could lead to a drastic overestimate in ecosystem response. Recognizing these patterns could help improve predictability of long-term plant responses. Indeed several examples from the recent literature point to such shifts in community structure driven by one factor diminishing the ecosystem response to another (Table 1) indicating that capturing these types of interactions in models will help predict future response of ecosystems to global change.

## Predicting the Global CO_2_ Fertilization Effect

A decade ago, global climate models systematically overestimated the ameliorating effects of terrestrial ecosystems on the future carbon cycle by excluding the nutrient limitation of the CO_2_ fertilization effect. Several more recent models have now incorporated this stoichiometric limitation (Sokolov *et al.* 2008; Thornton *et al.* 2009; Bala *et al.* 2013). Interestingly though, as models refine representation of the N cycle, some show that N may become less limiting in the future, owing to accelerated mineralization in response to warming and projections of increasing N deposition. Accounting for enhanced N availability, therefore, partially restores the large CO_2_ fertilization effect (Sokolov *et al.* 2008; Thornton *et al.* 2009; Zaehle and Dalmonech 2011). Evidence from long-term CO_2_ studies suggests that N scarcity limits the CO_2_ stimulation of productivity in temperate ecosystems (Norby *et al.* 2010; Reich and Hobbie 2012). However, N addition exerts a strong influence on plant diversity and species composition (Tilman 1987; Isbell *et al.* 2013) that must be considered when extrapolating to a future with widespread and heterogeneous increase in N availability.

It has been suggested that Earth System models, many of which represent biodiversity coarsely, overestimate the negative effects of global change on ecosystem processes because they exclude the mitigating effects of plant species shifts (Purves and Pacala 2008) apparent in diversity experiments (Tilman *et al.* 2006). For instance, higher plant biodiversity diminished the negative effects of drought on plant productivity (Tilman *et al.* 2006; Reich 2009). However, in predicting an enhanced terrestrial carbon sink in the future (Pinsonneault *et al.* 2011; Bala *et al.* 2013), the expectation is reversed—rather than relying on *stable* ecosystem functioning, society stands to benefit from a *dynamic* ecosystem function, specifically, increasing net carbon uptake through time. If this dramatic change in ecosystem behaviour does not persist, then the atmospheric CO_2_ concentration will rise more than expected, and climate change will be more severe than presently predicted.

## Conclusions

We have focused on how the interactive effects of two added resources may affect plant communities to drive ecosystem function. Yet, the recognition of community shifts in global change response applies to other factors besides enhanced resource availabilities (De Marco *et al.* 2013). For instance, if ozone tolerance were negatively related to CO_2_ responsiveness across plant taxa, then ozone exposure will favour species that exhibit a slighter CO_2_ response, thereby dampening the ecosystem-level CO_2_ response. The combination of CO_2_ and N could likewise favour a unique subset of species that can take advantage of the combination of high CO_2_ and high N. For example, some invasive species have been shown to respond more strongly to both elevated CO_2_ and N enrichment than non-invasives (Dukes *et al.* 2011; Mozdzer and Megonigal 2012). This general propensity for invasives to take advantage of high resource availability (Davidson *et al.* 2011) may indicate that they are not as constrained by the same tradeoffs as other plants. The degree to which community will shift and ecosystem productivity will respond to future change will partly depend on the degree to which individual plant responses to global change drivers covary across taxa.

## Sources of Funding

J.A.L. was supported by the National Science Foundation's Long-Term Research Environmental Biology Program (DEB-0950080). B.A.H. was supported by NSF (DEB-0949460).

## Contributions by the Authors

J.A.L. and B.A.H. wrote the manuscript.

## Conflicts of Interest Statement

None declared.
